# Induction of glutathione and flavonoid biosynthesis activates antioxidant enzymes to enhance drought tolerance in rice

**DOI:** 10.1186/s12870-026-08448-3

**Published:** 2026-03-09

**Authors:** Biru Alemu Chali, Yunfang Li, Mohammad Abass Ahanger, Phyu Phyu Thin, Jinpeng Wan, Peng Xu

**Affiliations:** 1 Key Laboratory of Tropical Plant Resource and Sustainable Use, Xishuangbanna Tropical Botanical Garden, Chinese Academy of Sciences, Mengla, Yunnan 666303 China; 2 University of the Chinese Academy of Sciences, Beijing, 101408 China; 3 Department of Plant Biotechnology, Bio and Emerging Technology Institute, Addis Ababa, Ethiopia

**Keywords:** Drought tolerance, Metabolome, Transcriptome, Antioxidant enzymes, Glutathione

## Abstract

Drought stress during early seed germination severely impacts rice establishment and productivity, yet the molecular and metabolic mechanisms underlying drought tolerance at this stage remain poorly understood. In this study, Luying 46 (LY46), a drought-tolerant genotype, and Haogenai (HGN), a drought-sensitive genotype, were used to investigate key adaptive traits and regulatory networks that confer drought resilience during germination. LY46 showed higher germination rates, better shoot and root growth under drought stress compared to HGN. Integrated transcriptomic and metabolomic analyses revealed enhanced glycolysis, glutathione metabolism, and phenylpropanoid biosynthesis as central pathways supporting drought adaptation in LY46. Upregulation of genes involved in starch mobilization and sugar metabolism facilitated energy production under water deficit, while elevated antioxidant enzyme activities, flavonoids, phenolic acid and prolines, mitigated oxidative damage. Metabolite profiling further revealed accumulation of peptides, amines, nucleotides, alkaloids, lipids and fatty acids in LY46 under drought. Weighted gene coexpression network analysis (WGCNA) revealed the coordinated regulation of antioxidant, osmoprotectant, and metabolic pathways involved in alleviating drought stress. Biochemical assays confirmed LY46’s maintenance of membrane integrity by effectively reducing lipid peroxidation under stress. Collectively, our comprehensive multiomics approach revealed a coordinated molecular and metabolic network regulated by transcription factors that underpins drought tolerance during rice germination, providing valuable insights into drought resilience in rice.

**Supplementary Information:**

The online version contains supplementary material available at 10.1186/s12870-026-08448-3.

## Introduction

As sessile organisms, plants encounter numerous challenges, including abiotic stresses such as drought, high temperature, and high salinity, as well as biotic stresses such as pathogen infection and insect injuries. These stresses affect plant growth, development and ultimately productivity. Among them, drought stress is one of the most critical factors affecting crop productivity. Water scarcity, as one of the main limiting factors for sustainable agriculture, has become a widespread phenomenon worldwide due to global climate change. The agriculture sector alone is responsible for 70% of global freshwater withdrawals and over 90% of its consumptive use from both surface and groundwater sources. Although the agriculture sector has significant potential to adjust its water usage, this must be achieved amidst rising food demand driven by population growth and shifts in dietary preferences [1].

Given the impact of drought on crop productivity, especially in staple crops like rice, advancing our understanding and improving drought tolerance are essential to ensure global food security. Rice, which typically grows in optimal environments, experiences a significant yield reduction under drought conditions compared to other crops. Annually, drought affects nearly 50% of rice production [2]. Recent research indicates that drought stress can reduce yields up to 90%, depending on its severity, duration, and growth stage of rice [3]. The early seed germination and seedling stages constitute a fundamental period that shapes crop establishment and ultimate productivity, particularly in direct dry-seeded rice where seeds face direct exposure to dry soil and water stress. Thus, it is imperative to understand and enhance drought stress tolerance at this stage to ensure food security [4].

Plants respond to environmental stress through complex physiological and molecular mechanisms, including antioxidant enzyme activation, cellular osmotic balance maintenance, hormone modulation, compatible solutes accumulation and various stress-responsive genes, all contributing to crop survival and adaptation [5]. Proline accumulation, a compatible solute Numerous studies have shown the accumulation of proline, a key compatible solute, plays a central role in stress tolerance [3], while antioxidant enzymes such as superoxide dismutase (SOD), peroxidase (POD), catalase (CAT), and glutathione S-transferase (GST), together with antioxidants such as flavonoids and phenolics mitigate oxidative damage by neutralizing reactive oxygen species [6].

Previous studies also revealed japonica varieties exhibit lower levels of antioxidant metabolites compared to indica ecotypes [7]. Integrative metabolomic and transcriptomic analysis have highlighted the role of *OsCIPK17* (*LOC_Os05g04550*) in enhancing sugar accumulation, thereby alleviating osmotic stress [8]. Key metabolic pathways such as citrate cycle, pentose phosphate pathway, glycolysis, and L-phenylalanine biosynthesis play essential roles in supporting drought tolerance [9].

Transcription factors (TFs) intricately regulate these defense mechanisms, serving as a master switch in drought response networks. In rice, activation of TFs such as *OsDREB1*, *OsWRKY11*, *OsNAC25*, and *OsBZIP72* enhances drought tolerance via activation of stress-responsive pathways [10–13]. Conversely, deactivating certain TFs such as *OsWRKY5*, *OsWRKY12*, *OsWRKY114*, and *OsBZIP52* has also been shown to improve drought tolerance [14–17]. These contrasting roles of TFs highlighted the dynamic nature of transcriptional regulation based on specific conditions, further underscoring the need for integrative transcriptome–metabolome analyses to unravel the complex regulatory networks that coordinated plant responses and adaptation to water deficit. Over the past decades, substantial progress has been made in delineating drought-responsive transcriptional factor networks, with DREB/CBF, NAC, MYB, WRKY, bZIP, and HD-Zip identified as key regulatory cascades [18]. Previous studies revealed the dynamic molecular and metabolic changes regulated by TFs during the early seed germination stage under optimal conditions [19–22]; however, such studies under optimal conditions provide insufficient insights into how seeds respond when exposed to stress. To address this, we conducted a comprehensive analysis integrating physiological, transcriptomic, and metabolomic profiling of two rice varieties, LY46 and HGN, under both optimal and PEG-induced stress. Previous studies have identified 15% PEG-6000 as the optimal level for differentiating genotypes under drought stress [23]. Screening for osmotic stress tolerance revealed that, despite both being upland types, HGN is highly sensitive compared to LY46.

Our study revealed distinct transcriptional responses and uncovered metabolism-related adaptation mechanisms underlying LY46’s superior PEG-induced osmotic stress tolerance during early germination stages compared to inferior HGN. PEG-induced osmotic stress caused significant changes in metabolite profiles of both genotypes, with key stress-related compounds including flavonoids, phenolic acids, alkaloids, nucleotides, peptides, lipids, fatty acids and amines accumulating to a greater extent in LY46 than in HGN. Furthermore, glutathione and flavonoid metabolic pathways, governed by transcriptional regulatory networks, played a crucial role in modulating antioxidant enzyme activity under drought stress in LY46. This study highlighted the molecular and metabolic mechanisms of osmotic stress tolerance during the early seed germination stage, providing a valuable foundation for future research aimed at improving dry direct-seeded rice productivity under drought stress.

## Materials and methods

### Experimental conditions and plant materials

Two genotypes, LY46 and HGN, that showed a contrasting pattern of drought tolerance were selected. LY46 is an upland rice variety introduced from International Rice Research Institute by Yunnan Academy of Agricultural Sciences and approved by Yunnan Province, while HGN is a landrace collected from Xishuangbanna, China. Both genotypes are maintained at the breeding base of Xishuangbanna Tropical Botanical Garden. The selected seeds were surface sterilized using a 10% sodium hypochlorite solution for 10 min (min), followed by thorough rinsing with deionized water (ddH_2_O) three times. Fifteen seeds, considered as one replication, were arranged on filter paper within 9 cm diameter petri dishes, and three replications for each treatment were conducted. The seeds from each accession were divided into two categories: the control group and the polyethylene glycol (PEG-6000) group. Each petri dish received 10 mL of ddH_2_O for the control group and 10 mL of 15% PEG-6000 (w: v) for the treatment group. The seeds were then germinated for seven days at a temperature of 28 °C under 16/8 h light/dark photoperiods in a plant growth chamber.

### Assessment of morphological parameters

The number of germinated seeds was recorded daily until the seventh day. A seed was considered germinated when its embryonic root or shoot reached half the length of the seed. On the seventh day, root number, root and shoot length were measured. The germination rate (GR) was calculated as a proportion of germinated seeds out of the total seeds. Germination efficiency percentage was assessed according to a designated formula [24].

### Assessment of physiological parameters

To analyze physiological parameters, seeds were collected at 1, 3, and 5 days after imbibition, with each time point sampled in three replicates. Lipid peroxidation was assessed by measuring malondialdehyde content (MDA). Briefly, 0.1 g seed sample was ground in liquid nitrogen, homogenized with 3 mL of 100 mM phosphate buffer solution (PBS) buffer (pH 7.8) and centrifuged at 10,000 x *g* for 20 min at 4 °C. Then, 100 µL of the supernatant was mixed with 1 mL of 0.25% thiobarbituric acid (TBA) solution and boiled for 15 min. After cooling for 5 min on ice, absorbance was measured at 532 nm and 600 nm using a UV spectrophotometer. A solution consisting of 1 mL of 0.25% TBA solution with 100 µL of 100 mM PBS (pH 7.8) was used as a reference [25].

The free proline content was determined using a defined procedure [26]. Briefly, a 0.1 g sample of seeds was extracted in 5 mL of 3% (w/v) sulphosalicylic acid, followed by centrifugation at 3000 g for 10 min. Two milliliters of the supernatant were mixed with 2 mL glacial acetic acid and 2 mL acid ninhydrin, then incubated for 1 h at 100 °C. The reaction was halted on ice, and free proline was extracted using toluene in a separating funnel, with optical density measured at 520 nm.

Total soluble sugars were determined using the anthrone method. One hundred milligrams of the powdered sample were extracted in 5 mL of 80% ethanol, followed by centrifugation at 5000 *g* for 10 min. About 0.5 mL of the supernatant was taken, and the volume was adjusted to 1 mL with ddH_2_O. One milliliter of 1 N HCl was added, and the mixture was heated in a boiling water bath. Subsequently, 4 mL of 0.2% anthrone was added, and the mixture was incubated in the water bath for 10 min. The optical density was recorded at 620 nm [27].

To analyze phenolic compounds, 0.1 g of powdered seed was extracted in 2mL 80% ethanol and centrifuged at 10,000 g for 20 min. The supernatant was evaporated to dryness and dissolved in 5 mL of distilled water. An aliquot of 0.5 mL was made up to 1 mL with distilled water, followed by the addition of 1mL Folin-Ciocalteu’s reagent (1 N). After a 3 min incubation, 2 mL of 20% Na_2_CO_3_ was added, and the mixture was heated in a boiling water bath for 1 min, cooled, and the absorbance was measured at 650 nm against a blank using a spectrophotometer. Concentration of phenols was expressed in mg. g^− 1^ fresh weight, equivalent to catechol [28].

Total flavonoid content was determined by a colorimetric method [29] with minor modification. Briefly, 0.1 g of powdered seed was extracted in 3 mL of methanol and centrifuged at 10,000 *g* for 10 min. Thereafter, 0.5 mL of the extract solution was diluted with 0.5 mL of distilled water and mixed with 2 mL of 5% NaNO_2_ and incubated for 5 min. After 5 min, 2 mL of 10% AlCl₃ was added, followed by another 5 min of incubation. Subsequently, 1 mL of 0.1 mM NaOH was added and incubated for 15 min in the dark. The absorbance was measured at 510 nm against a blank, and the calculation was done using a standard curve of catechin.

Fresh plant samples (0.1 g) were homogenized in chilled PBS (pH 7.5) with 0.1% PVP and 0.5 mM EDTA, then centrifuged at 12,000 *g* for 15 min. The supernatant was used as the enzyme source. For guaiacol peroxidase, a reaction solution was made by mixing 28 µL of 0.2% guaiacol with 50 mL of 100 mM PBS (pH 7), heated and cooled, then 19 µL of 30% H_2_O_2_ was added. In a cuvette, 50 µL enzyme extract was mixed with 1 mL reaction solution. A reference contained 50 µL PBS and 1 mL reaction solution. Absorbance was measured at 470 nm every 15 s for 1 min [25]. For glutathione S-transferase, a reaction solution was made by combining 20 mL of 5 mM GSH with 1.5 mM CDNB. In a cuvette, 50 µL of enzyme extract was added to 1 mL of the reaction solution. Controls included 1 mL reaction solution with 50 µL of 100 mM PBS (pH 7.8), while ddH_2_O was used as a reference. The absorbance was recorded at 340 nm by a UV spectrophotometer every 15 s for 1 min [25]. Superoxide dismutase activity (SOD) (EC 1.15.1.1) was measured according to the previously described methods with a minor modification [30]. Briefly, the assay mixture contained 1.5 mL of PBS (pH 7.8), 0.2 mL of methionine, 0.1 mL of enzyme extract, 1 M Na_2_CO_3_, 2.25 mM nitro blue tetrazolium (NBT), 3 mM EDTA, riboflavin, and 1 mL of ddH_2_O. After 15 min incubation under a 15 W fluorescent lamp, absorbance was measured at 560 nm. Two blanks were prepared: Blank A in the dark and Blank B in light without the enzyme extract. The percent color reduction between blank B and the sample was calculated, with a 50% color reduction defined as one unit of enzyme activity, expressed as EU mg^− 1^ protein.

### Sampling for transcriptome and metabolome

Samples were collected in three biological replicates for in-depth analysis of transcriptomic and metabolic profiling at 36 and 72 h (2 genotypes x 2 treatments x 2 time points x 3 biological replicates). These samples were immediately frozen in liquid nitrogen and stored at -80 °C for subsequent studies.

### Transcriptome and metabolome profiling

Metabolite profiling was performed as described previously [31]. LY46 and HGN seed samples collected at 36 and 72 h after imbibition (HAI) under control and PEG-6000 conditions by Wuhan Metware Biotechnology Co., Ltd. (Wuhan, China). Briefly, samples were freeze-dried using a Scientz-100 F freeze dryer and vacuum-frozen for 63 h. Subsequently, the seeds were ground into powder using a mixer mill (MM 400, Retsch) with a zirconia bead for 1.5 min at 30 Hz. Then, 50 mg sample powder was weighed using an electronic balance (MS105DM), and 1200 µL of -20 °C precooled 70% methanol, water internal standard extract was added and vortexed once every 30 min for 30 s each time, for a total of 6 times. Following centrifugation at 12,000 rpm for 3 min, the supernatant was aspirated, and the sample was filtered through a microporous filter membrane (0.22 μm pore size) and stored in a sample injection vial for UPLC-MS/MS analysis. Analyst software v.1.6.3 was used to process the data. Differentially expressed metabolites (DEMs) between groups were identified by the criteria of variable importance projection (VIP) ≥ 1 and Log_2_FC (Fold change) ≥ 1.

Transcriptome profiling was performed as described previously [32]. Briefly, total RNA from samples was extracted by ethanol precipitation and CTAB-PBIOZOL. After extraction, RNA was dissolved by adding 50 µL of DEPC-treated water. Subsequently, total RNA was identified and quantified using a Qubit fluorescence quantifier and a Qsep400 high-throughput bio fragment analyzer. Raw data were processed using FASTP to remove reads containing adapters, reads with over 10% ambiguous bases, and reads where more than 50% of bases had low quality (Q ≤ 20). Subsequent analyses were performed using clean reads. Clean reads were aligned to the reference genome IRGSP-1.0 using HISAT2. Gene alignment statistics were calculated using feature Counts with paired-end counting, followed by the computation of fragments per kilobase million (FPKM) values for each gene to estimate gene expression levels. Differential gene expression (DEGs) analysis was performed using DESeq2 by applying the Benjamini & Hochberg correction to *p*-values.

### Data analysis

Morphological data figures were generated by OriginLab (OriginLab, Northampton, USA). The mean ± standard deviation (SD) was calculated, and *t*-tests were performed with significance levels indicated as *p < 0.05, **p < 0.01, and ***p < 0.001. Physio-biochemical traits of rice genotypes were evaluated using ANOVA and Fisher’s LSD test was used to separate treatment means (*p* < 0.05). To compare gene and metabolite profiles between genotypes and time points, principal component analysis (PCA) and hierarchical clustering analysis were conducted using the procomp function and the ComplexHeatmap package in R (www.r-project.org). Enrichment analyses were performed using the Kyoto Encyclopedia of Genes and Genomes (KEGG), with pathways having a *p*-value < 0.05 considered significantly enriched. Additionally, WGCNA was utilized to explore gene relationships.

## Results

### Morphological responses of rice to PEG-induced drought stress

**Fig. 1:**
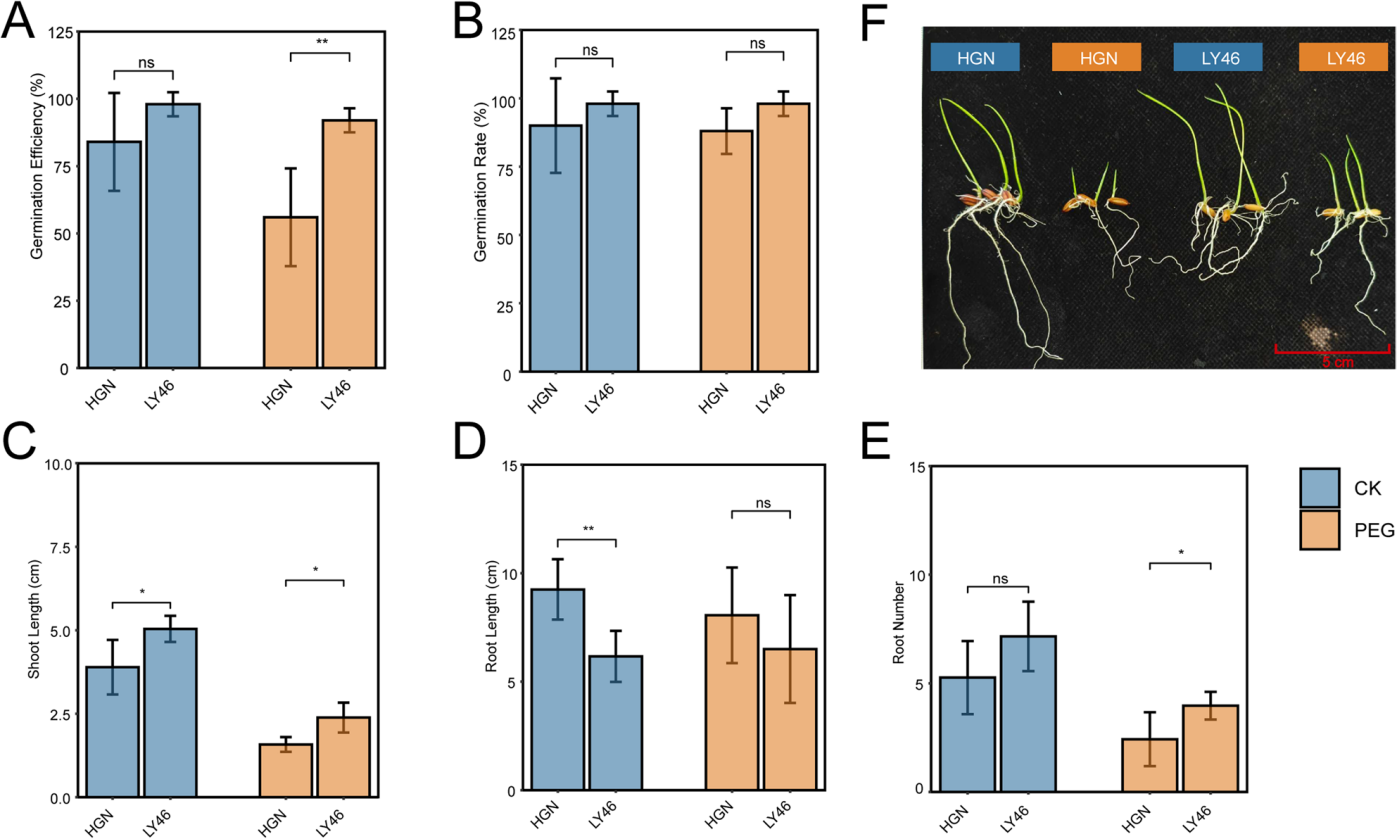
LY46 exhibits enhanced drought tolerance. **A-F** Germination efficiency (**A**), Germination rate (**B**), Shoot length (**C**), Root length (**D**), and Root number (**E**). Error bars indicate mean ± SD (*n* = 3). Asterisks mean significant difference between LY46 and HGN (based on *t*-test: *p < 0.05, **p < 0.01 and ***p < 0.001). **F** Representative phenotype of LY46 and HGN under CK and PEG conditions

LY46 exhibited better germination efficiency and germination rate compared to HGN under PEG-induced stress (Fig. 1, A and B). Shoot length showed a clear distinction between the two genotypes, with LY46 maintained robust growth under stress conditions, whereas HGN experienced a significant reduction in shoot length under stress (Fig. 1C). Root length was higher in HGN under control conditions, while it remains not significantly different from LY46 under stress (Fig. 1D). Conversely, LY46 produced a higher number of roots under stress conditions (Fig. 1E). These results indicated that LY46 performed superior tolerance to HGN.

To determine whether the differential responses of the two genotypes emanated from external barrier blocking water intake, SEM was used to examine seed coat permeability. SEM revealed structural differences with HGN exhibited a loose block-like structure with smoother granular structures between cells and no trichomes, while LY46 showed elongated, rigid structures with sharp trichomes protuding from regularly arranged cells (Fig. S1, A and B). To assess whether these differences affect water absorption, water uptake was tested under control and PEG-6000 conditions from 6 to 48 h after imbibition. Results showed higher water uptake in HGN under both conditions, indicating that its loose seed coat structure facilitates water absorption and that its drought sensitivity is not due to an external barrier (Fig. S1C).

### Physiological responses of rice to PEG-induced drought stress

LY46 consistently showed increased activities of key antioxidant enzymes such as superoxide dismutase, guaiacol peroxidase, and glutathione *S*-transferase under PEG treatment, along with elevated proline and phenol contents (Fig. S2). In contrast, HGN exhibited reduced antioxidant enzyme activities, decreased proline and soluble sugar levels, and a marked increase in malondialdehyde content, reflecting greater lipid peroxidation and cellular damage (Fig. S2). The flavonoid content in both genotypes showed a slighter change under stress (Fig. S2). Overall, LY46 demonstrates a more robust and adaptive biochemical response to drought stress than HGN.

### LY46 and HGN showed distinct transcriptomic alterations in response to drought

**Fig. 2:**
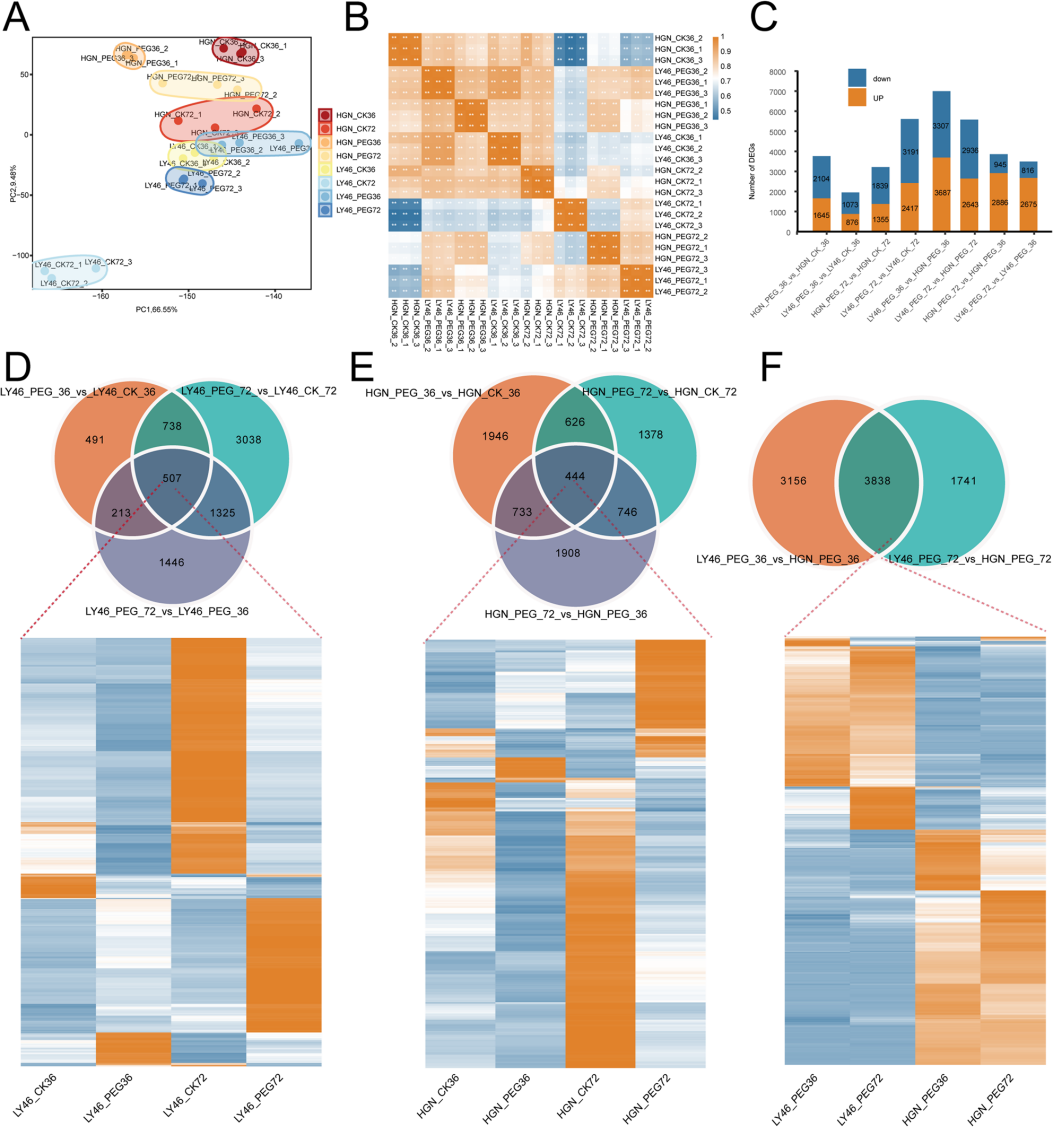
Transcriptomic analysis of LY46 and HGN under different drought durations. **A** Principal component analysis of all samples. **B** Sample correlation analysis. **C** Stacked bar plot of differentially expressed genes; The orange and blue colors indicate upregulated and downregulated genes, respectively. **D**–**F** Venn diagrams of DEGs in different comparisons and heatmaps of shared DEGs; The orange and blue colors indicates up- and downregulated genes

Transcriptome analysis of the two rice genotypes under control and PEG treatment generated over 174.99 Gb of clean reads from 24 libraries. Each sample yielded at least 5 Gb of clean reads, with an average Q30 base quality of 93.95%. Over 1.11 billion reads were successfully mapped to the reference genome (Tab. S1). Principal component analysis (PCA) distinctly separated the two genotypes along PC1 and PC2, explaining 66.55% and 9.48% of the total variation, respectively (Fig. 2A). This separation highlighted a clear transcriptomic difference between drought tolerant and drought sensitive genotypes under both treatment and across time points (Fig. 2A). Correlation analysis confirmed reproducibility among biological replicates (Fig. 2B).

In LY46, 2,675 DEGs were upregulated and 816 downregulated at 72 h compared to 36 h under PEG stress. Additionally, 876 DEGs were upregulated and 1,073 downregulated at 36 h under PEG *versus* control conditions, while 2,417 were upregulated and 3,191 downregulated at 72 h (Fig. 2C). In HGN, 2,886 DEGs were upregulated and 945 downregulated at 72 h compared to 36 h under PEG stress. Moreover, 1,645 DEGs were upregulated and 2,104 downregulated at 36 h under PEG *versus* control, while 1,355 were upregulated and 1,839 downregulated at 72 h (Fig. 2C). At 36 h after PEG treatment, 6,994 DEGs were identified between LY46 and HGN, with 3,687 upregulated and 3,307 downregulated. By 72 h, this number decreased to 5,579 DEGs, including 2,643 upregulated and 2,936 downregulated (Fig. 2C). Comparison of HGN at 36 and 72 h post-treatment revealed 444 shared DEGs, of which 147 were upregulated and 297 downregulated. The highest number of upregulated genes in HGN was observed under control conditions at 72 h, followed by PEG treatment at 72 h (Fig. 2E). Similarly, LY46 showed 507 shared DEGs at these time points, with 211 upregulated and 296 downregulated. LY46 had more upregulated genes under control conditions at 72 h, followed by PEG treatment at 72 h (Fig. 2D). When comparing DEGs between LY46 and HGN under stress at both time points, 3,838 shared DEGs were found, with 1,677 upregulated and 2,161 downregulated. Compared to HGN, LY46 showed more upregulated genes under stress at both time points (Fig. 2, D and E).

**Fig. 3:**
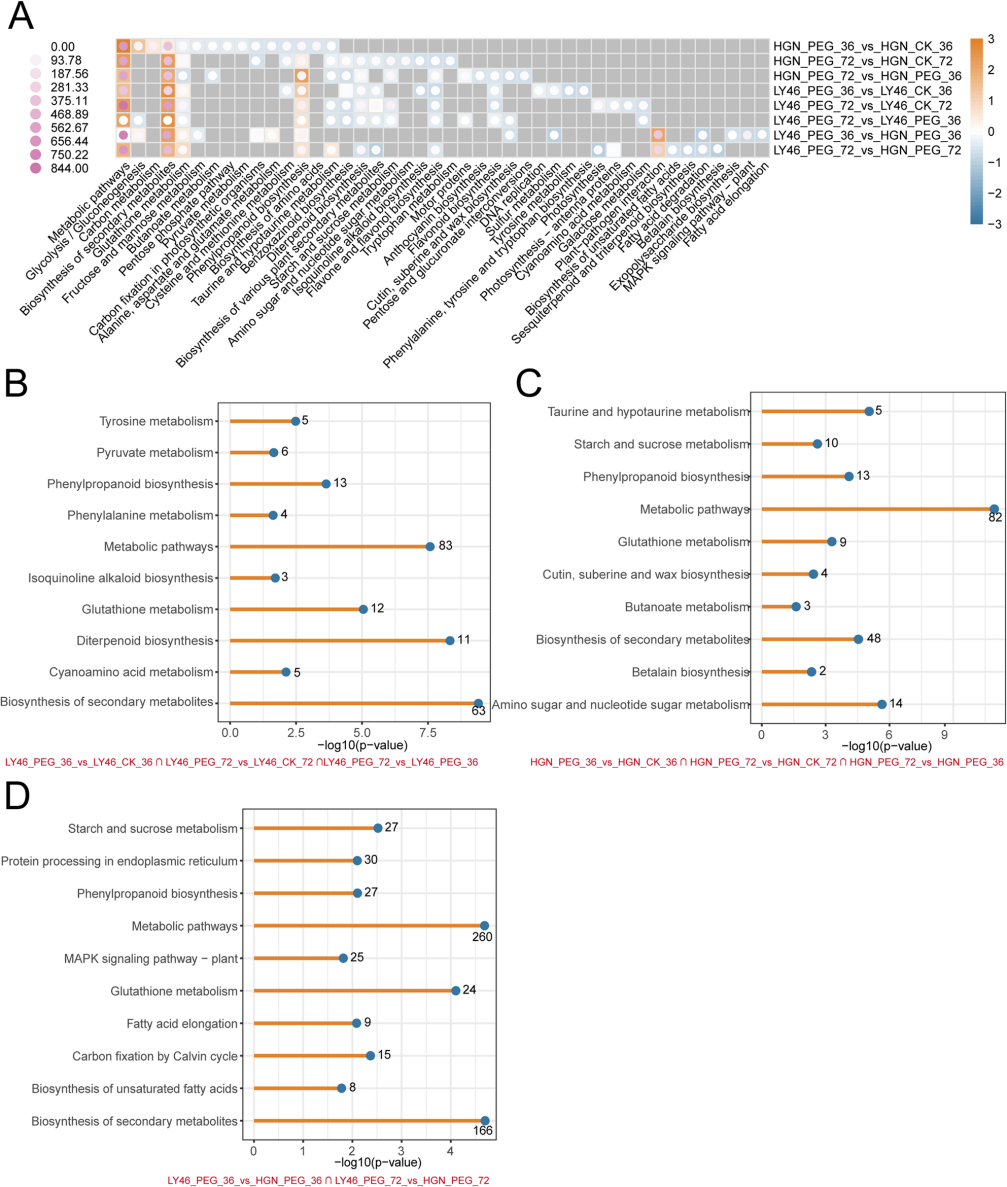
KEGG pathway enrichment analysis of LY46 and HGN under different drought durations. **A** Heatmap of the top 10 enriched KEGG pathways across all groups; color bars represent normalized *p*-values, with orange indicating lower *p*-values and blue indicating higher *p*-values. Dot size indicates the number of genes enriched in each pathway, and purple shades indicate greater gene enrichment. **B**-**D** The top 10 commonly enriched pathways among different groups.

KEGG pathway enrichment analysis was employed to explore the potential functions of DEGs and the core drought-responsive genes in the two genotypes under drought stress at 36 and 72 h. The results showed both genotypes shared enrichment in metabolic pathways, phenylpropanoid biosynthesis, and glutathione metabolism though the extent and significance of gene enrichment varied (Fig. 3A). The enrichment analysis in LY46 and HGN under stress revealed that both genotypes share major pathways like metabolic pathways, biosynthesis of secondary metabolites, glutathione metabolism, and phenylpropanoid biosynthesis that contribute drought stress tolerance (Fig. 3, B and C). Similarly, shared genes induced under drought stress in LY46 at both time points showed enrichment in metabolic pathways, phenylpropanoid biosynthesis, and glutathione metabolism (Fig. 3D).

**Fig. 4:**
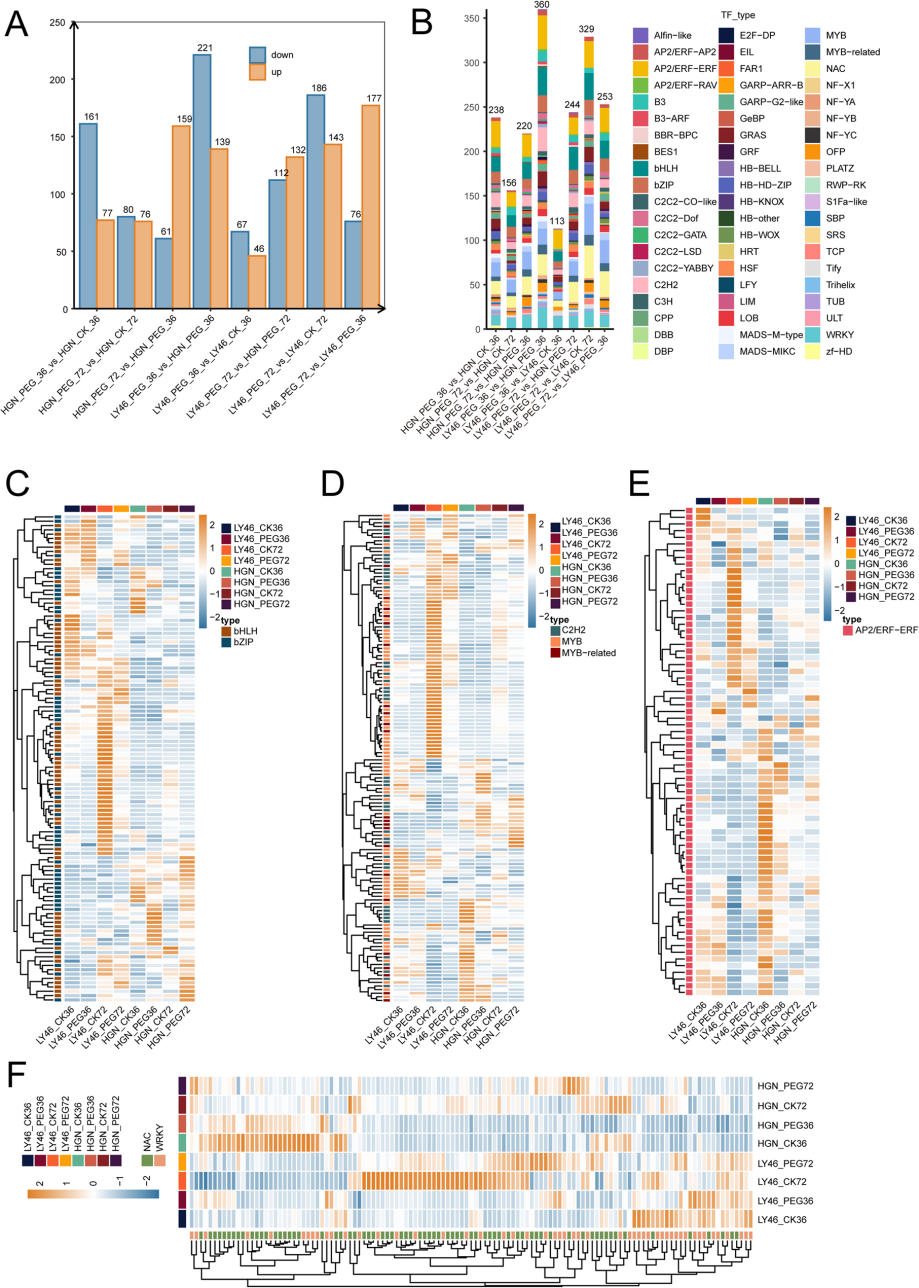
Differential transcription factor analysis and expression profiles of major TFs. **A** Bar plot showing the number of differentially expressed transcription factors. **B** Stacked bar plot of TF classification. **C**-**F** Heatmaps of commonly expressed transcription factors. The orange color indicates upregulated genes, and blue color indicates downregulated genes

TFs play a vital role in enabling plants to adapt and acclimate to various stresses [33]. We analyzed TFs regulating the drought response during the early imbibition stage in rice (Tab. S4). In LY46, a total of 177 TFs were upregulated, and 76 genes were downregulated at 72 h vs. 36 h under PEG. Comparing PEG to control, 113 TFs were found at 36 h and 329 at 72 h (Fig. 4A). In HGN, 159 TFs were upregulated, and 61 were downregulated at 72 h vs. 36 h under PEG. Similarly, comparing PEG to control, 238 TFs were found at 36 h and 156 at 72 h (Fig. 4A). Under PEG treatment at 36 h, 560 TFs were identified between LY46 and HGN, with 139 upregulated and 221 downregulated. By 72 h, TFs decreased to 244, including 132 upregulated and 112 downregulated (Fig. 4A). Several key TFs families, including AP2/ERF, BHLH, NAC, MYB, WRKY, and BZIP were highly expressed differentially in both genotypes. LY46 exhibited strong transcriptional activation with numerous upregulated TFs, including MYB, C2H2, MYB-related, BHLH, and BZIP, particularly at 36 and 72 h. In contrast, HGN showed predominantly downregulated TFs (Fig. 4, C and D). At 72 h under control conditions, AP2/ERF TFs were highly expressed in both genotypes (Fig. 4E). NAC and WRKY TFs were highly expressed at 72 h in LY46, while in HGN, they were upregulated early but declined in later stages (Fig. 4F). Overall, the differential expression patterns of BHLH, BZIP, MYB, NAC, and WRKY TFs between the two genotypes showed a distinct regulatory network involved in drought stress response. LY46 exhibited dynamic and coordinated upregulation of multiple stress-responsive TF families, whereas HGN displayed a more subdued and limited transcriptional response.

### Metabolomic profiles of LY46 and HGN in response to drought stress

**Fig. 5:**
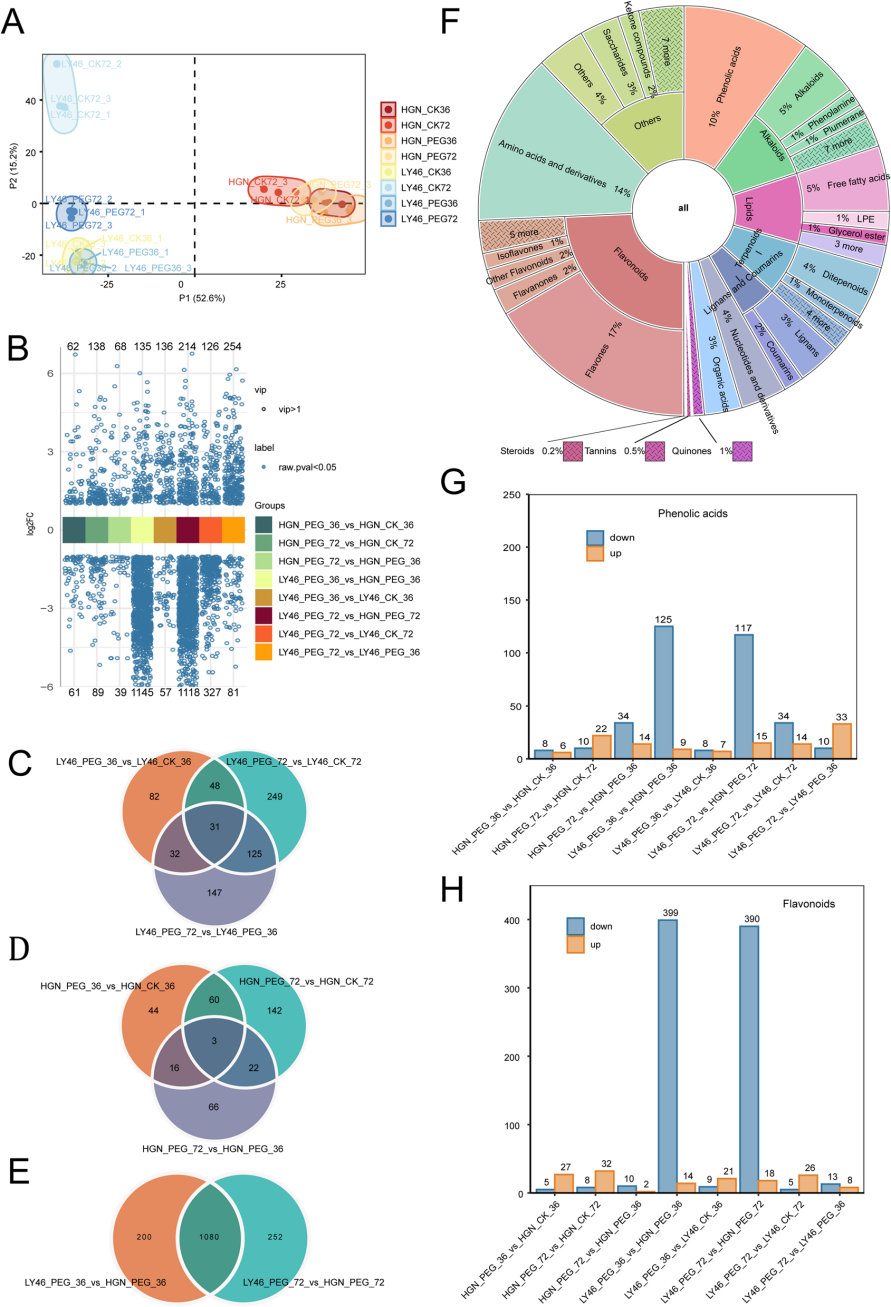
Metabolomic profiling of LY46 and HGN under different drought durations. **A** PLS-DA analysis of metabolite profiles. **B** Volcano plots of differentially expressed metabolites. **C**–**E** Venn diagrams of differentially expressed metabolites among comparison groups. **F** Classification pie chart of differential metabolites; inner and outer layers represent level 1 and level 2 classification, respectively. **G**–**H** Number of up- and downregulated phenolic acids and flavonoids. The orange and blue color indicated upregulation and downregulation

Metabolomics analysis identified 2,508 metabolites (Tab. S5). PCA effectively distinguished the two genotypes along PC1 and PC2, which accounted for 52.6% and 15.2% of the total variation, respectively (Fig. 5A). The data showed clear separation between LY46 and HGN genotypes under both treatment conditions and time points (Fig. 5A). The high reproducibility among the biological replicates was clearly observed (Fig. S3).

Differentially expressed metabolites (DEMs) were then analyzed to gain further insights into drought tolerance variation among the two genotypes. A total of 335, 193, and 453 DEMs were identified in LY46 under PEG stress at 72 h vs. 36 h, 36 h PEG vs. 36 h control, and 72 h PEG vs. 72 h control, respectively, with varying numbers of upregulated and downregulated DEMs (Fig. 5B). In HGN,107, 123, and 227 DEMs were identified across key PEG stress comparisons (Fig. 5B). Between LY46 and HGN, 1,280 DEMs were detected at 36 h post PEG stress, rising to 1332 by 72 h with more metabolites upregulated than before (Fig. 5C). Genotype specific comparisons revealed 3 common DEMs in HGN and 31 in LY46 across stress time points, while 1080 DEMs were shared between time points when comparing the two genotypes under stress (Fig. 5, C, D and E). Differential metabolite analysis revealed that flavonoids constituted the largest proportion (22%) of detected metabolites, followed by amino acids and derivatives (14%), phenolic acids (10%), and alkaloids (7%) (Fig. 5F).

A further phenolic acid analysis depicted both upregulation and downregulation across multiple comparisons. In HGN, 14, 32, and 48 DEMs were identified when comparing 36 h PEG vs. control, 72 h PEG vs. control, and 72 h vs. 36 h PEG treatments, respectively (Fig. 5G). Similarly, LY46 showed 15, 48, and 43 DEMs across the same comparisons, highlighting dynamic changes in phenolic acid regulation under stress in both genotypes (Fig. 5G). Phenolic acid showed a significant change when comparing LY46 and HGN under PEG stress, with 134 DEMs at 36 h and 132 DEMs at 72 h, most of which were predominantly downregulated (Fig. 5G).

Similarly, flavonoid levels depicted varied regulation across genotypes and time points. In HGN, 32, 40, and 12 DEMs were identified when comparing 36 h PEG vs. control, 72 h PEG vs. control, and 72 h vs.36 h under PEG treatments, respectively (Fig. 5H). LY46 showed 30, 31, and 21 DEMs in the same comparisons as HGN. Comparing LY46 and HGN under PEG stress revealed 413 and 408 DEMs at 36 and 72 h, respectively (Fig. 5H). Overall, there is a contrasting pattern of flavonoids and phenolic acids regulation between the HGN and LY46 genotypes, in which a large number of DEMs were upregulated in the latter genotypes under induced stress, implying that these important plant-derived compounds may play a crucial role in differential stress responses in the two genotypes. 

### Metabolic pathway regulation in LY46 and HGN under drought stress

**Fig. 6:**
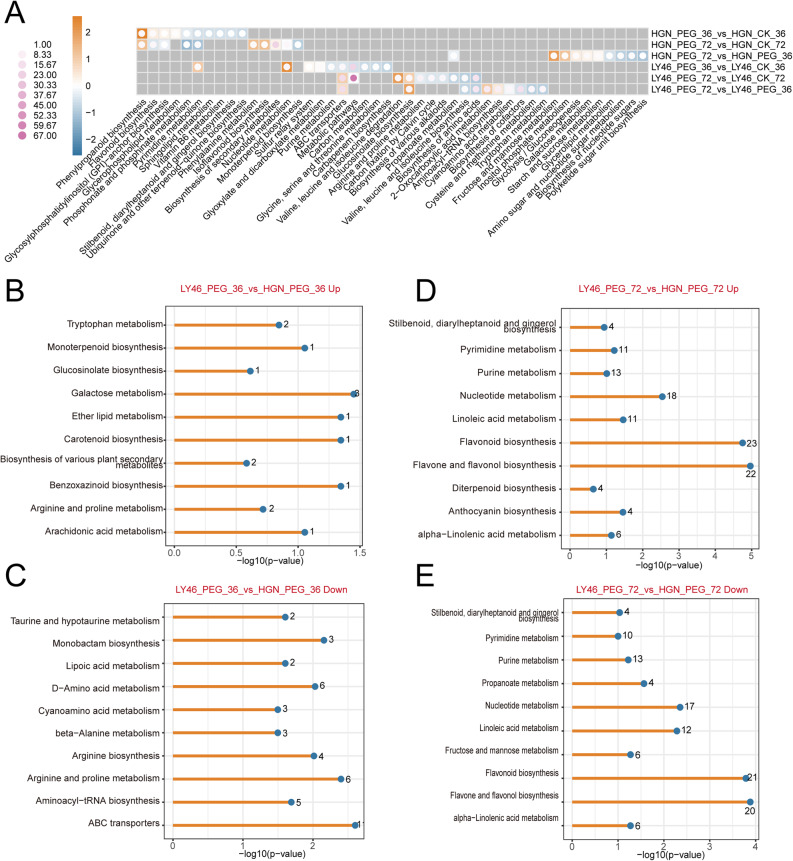
KEGG enrichment analysis DEMs in LY46 and HGN under different drought durations. **A** Heatmap of the top 10 enriched KEGG pathways in each group; color bars represent normalized *p*-values, with orange indicating lower and blue indicating higher *p*-values. Dot size represents the number of genes enriched per pathway; purple shades indicate greater gene enrichment. **B**–**C** Enriched KEGG pathways of up- and downregulated genes in LY46_PEG_36 vs. HGN_PEG_36. **D**–**E** Enriched KEGG pathways of up- and downregulated genes in LY46_PEG_72 vs. HGN_PEG_72

A comparative analysis of metabolic pathways under drought stress between HGN and LY46 at 36 and 72 h revealed a distinct DEMs enrichment. In HGN, pathways such as phenylpropanoid biosynthesis, phenylalanine metabolism, isoflavonoid biosynthesis, fructose and mannose metabolism and inositol phosphate metabolisms were upregulated, while phosphonate metabolism, pyrimidine metabolism, monoterpenoid biosynthesis, glycerolipid metabolism, and amino sugar and nucleotide sugar metabolism were downregulated (Fig. 6A). In LY46, DEMs enriched in pyrimidine metabolisms, nucleotide metabolisms, valine, leucine, and isoleucine degradation, glucosinolate biosynthesis and 2-oxocarboxylic acid metabolisms were upregulated, with downregulation in tryptophan metabolism, cysteine and methionine metabolism, biosynthesis of amino acids, and related pathways (Fig. 6A).

Further pathways analysis showed that LY46 and HGN employed different strategies to cope drought stress over time points. Early stages of stress exposure activated upregulation of galactose metabolism, tryptophan metabolism, arginine and proline metabolism, and carotenoid biosynthesis, while D-amino acid metabolism, aminoacyl-tRNA biosynthesis, arginine and proline biosynthesis, and ABC transporter pathways were downregulated (Fig. 6, B and C). As stress continued, flavonoid biosynthesis, nucleotide metabolism, and alpha-linolenic acid metabolism became upregulated, while fructose and mannose metabolism, propanoate metabolism, pyrimidine metabolism, and purine metabolism were downregulated. Interestingly, some pathways like flavonoid biosynthesis, nucleotide metabolism, and alpha-linolenic acid metabolism showed both up and downregulation, indicating that different components within these pathways can be regulated differently depending on specific cellular demands (Fig. 6, D and E). The coordinated upregulation and downregulation of key metabolic pathways during stress exposure orchestrate a sophisticated regulatory balance, allowing the plant to fine-tune their biochemical responses and adapt effectively to stress.

### Glycolysis and glutathione metabolism response to drought stress

**Fig. 7:**
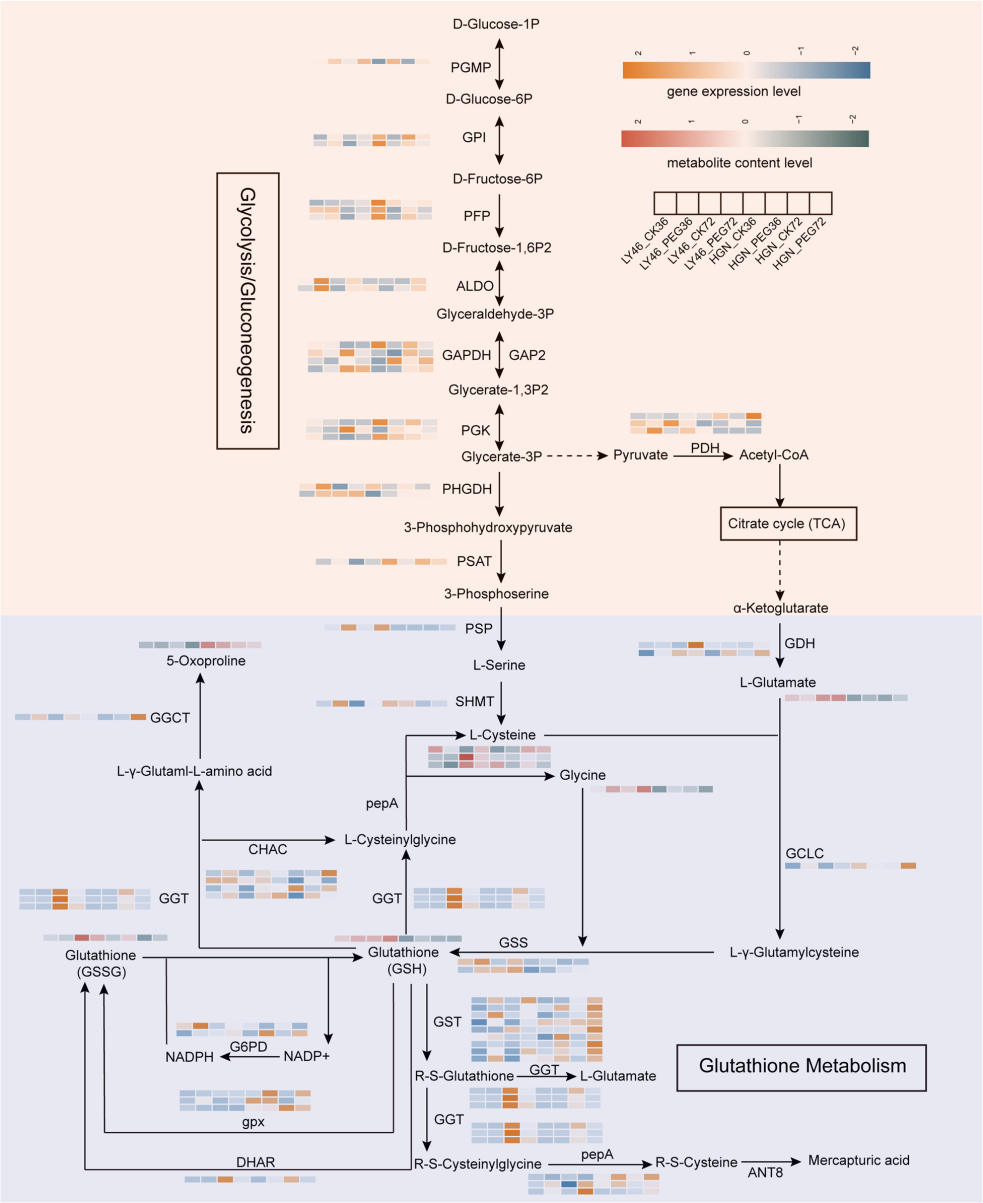
Response of the glycolysis/ gluconeogenesis and glutathione metabolism pathway to drought stress. The orange and blue colors indicate up- and downregulated gene expression, while red and gray colors indicate up- and downregulated metabolite accumulation

In the glycolysis/gluconeogenesis pathway, higher expression of genes encoding key enzymes such as PGMP, PFP, ALDO, GAPDH, and GAP2 were observed in LY46. Additionally, metabolites such as D-Glucose-6P, D-Fructose-1,6P, D-Fructose-1,6P2, Glycerate-1,3P2, and 3-phosphohydroxypyruvate were more abundant in LY46 under stress conditions. The enrichment of pyruvate metabolism in LY46 also suggests a better energy balance under drought stress (Fig. 7). Additionally, LY46 showed higher expression of genes encoding PHGDH, PSAT, and PSP enzymes that link glycolysis to serine biosynthesis, potentially impacting downstream sulfur amino acid (like cysteine) and glutathione production. Glycolytic gene expression and metabolite levels in HGN remained largely unaffected (Fig. 7).

In glutathione metabolism, the expression of genes encoding enzymes like GSS, GST, and GGT was elevated, which were crucial for glutathione synthesis, along with higher levels of glutathione (GSH), indicating a strong glutathione-mediated antioxidant response in LY46. Glutathione metabolism was less active in HGN samples, as evidenced by lower gene expression and reduced GSH metabolite content within this pathway. Overall, stronger glycolysis and glutathione metabolism was observed in LY46 than in HGN. 

### Flavonoid biosynthesis pathways response to drought stress

**Fig. 8:**
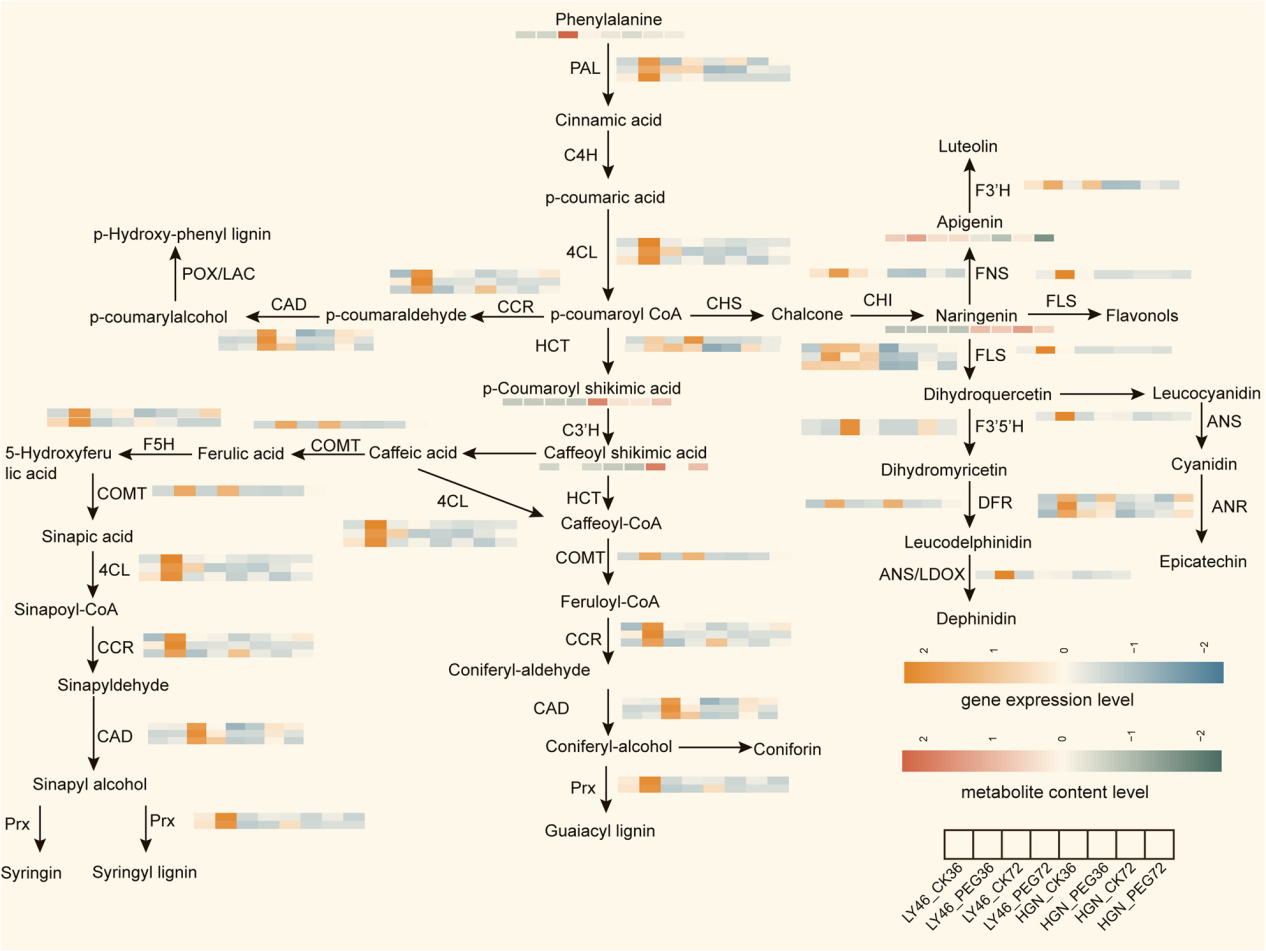
Response of the phenylpropanoid and flavonoid biosynthesis pathways to drought stress. The orange and blue colors represent up- and downregulated gene expression, and red and gray colors indicate up- and downregulated metabolite accumulation

LY46 exhibited higher expression levels of genes encoding PAL compared to HGN, especially under PEG treatment at 36 h, indicating enhanced activation of the initial steps of phenylpropanoid metabolism in LY46 (Fig. 8). Enzymes converting p-coumaric acid to p-coumaroyl CoA (4CL) and those involved in lignin biosynthesis (COMT, CCR, CAD, Prx) were more active in LY46, consistent with increased levels of lignin intermediates like coniferyl and sinapyl alcohols. Enzymes modifying ferulic acid (COMT, F5H) also have elevated expression in LY46, suggesting more active lignin formation in LY46 at early exposure to stress. In the flavonoid branch, genes encoding for flavonol and flavone synthesis (CHS, CHI, FLS, F3H) and related metabolites such as luteolin and apigenin were more abundant in LY46 under early PEG stress. In contrast, HGN generally shows lower gene expression and metabolite levels, except for higher naringenin and shikimic acid (Fig. 8). Overall, PEG treatment significantly boosts phenylpropanoid and flavonoid metabolism in LY46, reflecting a stronger metabolic response than the relatively slowly and quite changes in HGN.

### Coexpression network analysis reveals integrated regulatory mechanisms

**Fig. 9:**
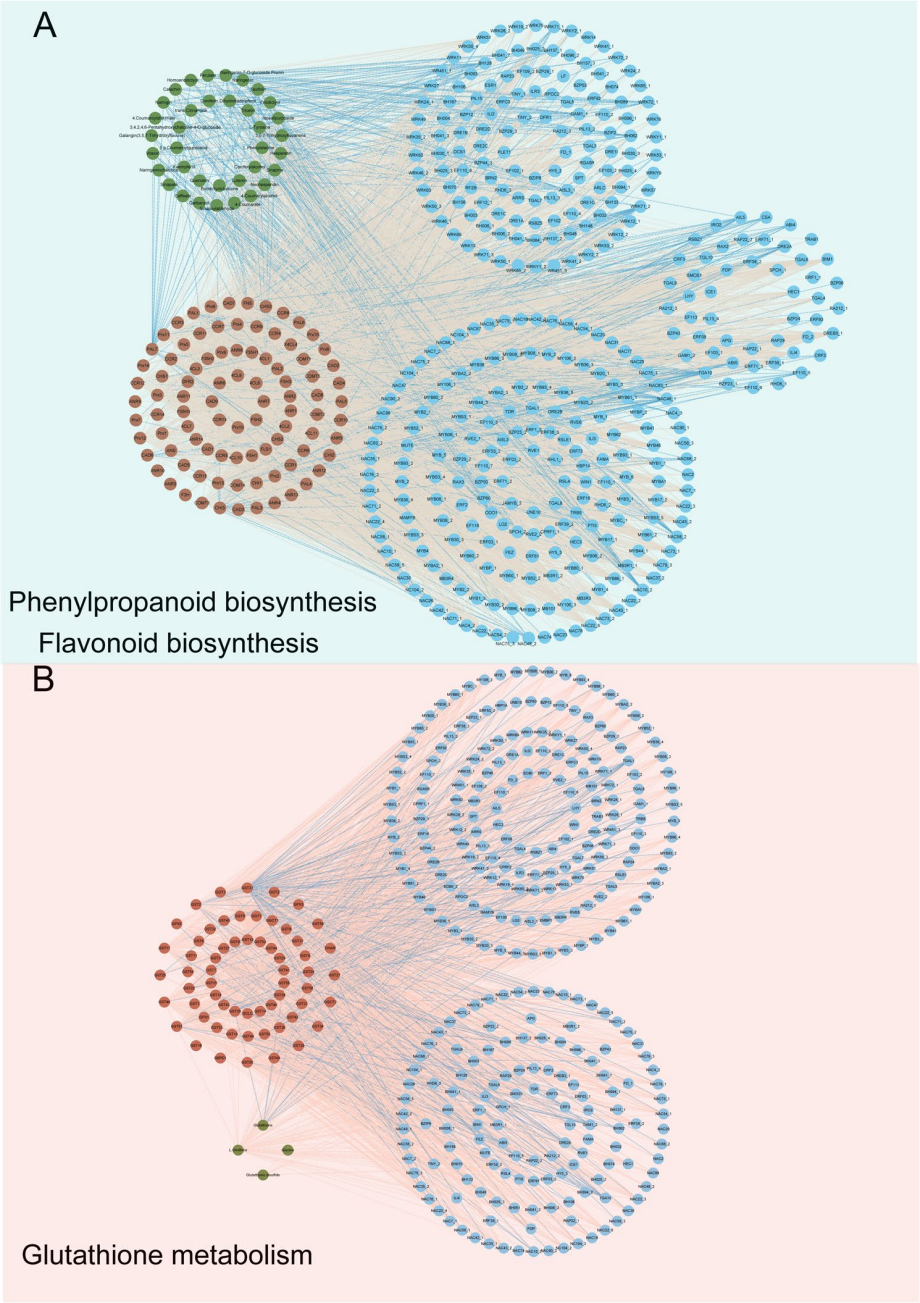
Co-expression network of genes, metabolites, and transcription factors involved in glutathione, flavonoid, and phenylpropanoid metabolism. Green, red and blue circles represent metabolites, genes, and blue key transcription factors, respectively. The network was constructed based on |*r*| > 0.8 and *p* < 0.05. Red dashed lines indicate positive correlations and blue dashed lines indicate negative correlations

The further co expression analysis of genes, metabolites, and TFs networks within glutathione, phenylpropanoid, and flavonoid biosynthesis pathways identified a highly interconnected network showing an intertwined regulatory complex. The phenylpropanoid and flavonoid biosynthesis network displayed a highly dense cluster connected to flavonoid metabolism genes, flavonoid-related metabolites and TFs, forming a distinct cluster with clear boundaries (Fig. 9A). Most TFs exhibited positive correlations with flavonoid metabolism genes, although some showed negative correlations (Tab. S7). This pattern suggests that TFs regulate specific subsets of genes dedicated to different branches of secondary metabolite biosynthesis, allowing plants to selectively produce flavonoids and phenylpropanoids in response to environmental stimuli such as exposure to PEG-induced drought stress.

In glutathione metabolism, a highly interconnected set of TFs and glutathione metabolism genes with fewer metabolites, with either positive or negative correlations, signifying a dynamic balance of activation and repression mechanisms (Fig. 9B). This activation and repression of glutathione metabolism play a crucial role in maintaining cellular redox homeostasis and detoxification by coordinating transcriptional responses to oxidative stress. Unlike flavonoid and phenylpropanoid metabolism, most TFs showed negative correlations with glutathione metabolism genes. However, *GST4*,* GST5* and *GST30* showed a positive correlation with *MYB*3, *WRKY12* and *WRKY51* (Tab. S6), highlighting that these TFs likely modulated gene expression to enable precise metabolic control in response to drought stress. Taken together, our data illustrated how plants employ transcriptional and metabolic strategies to regulate essential biochemical pathways, ensuring both survival and adaptation through complex molecular interactions. 

**Fig. 10:**
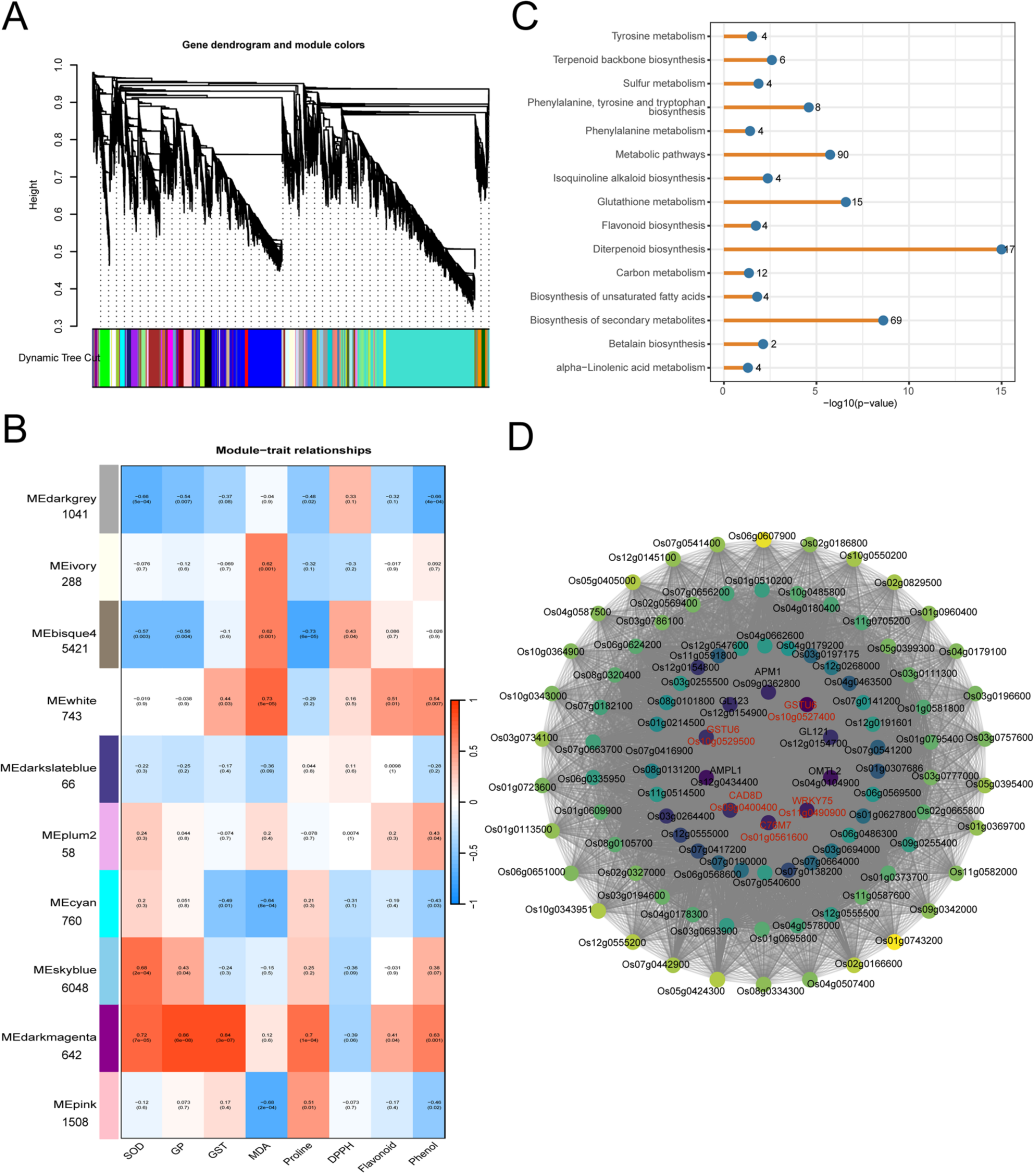
WGCNA-based identification of hub genes. **A**–**B** Dendrogram of module clustering and trait-associated modules. The red color indicates a positive correlation, and the blue color indicates a negative correlation, with *p*-values in parentheses. **C** Top 15 KEGG enrichment pathways of genes in the MEdarkmagenta module. **D** Gene co-expression network of the MEdarkmagenta module. The darker colors indicate higher connectivity within the module

WGCNA was then used to gain deeper insight into the regulatory mechanisms under drought stress by exploring coexpression networks of the DEGs, resulting identification of 10 coexpression modules based on gene expression similarities (Fig. 10A). Module trait analysis revealed a key association between groups of expressed genes (modules) and physiological traits. Genes in the white, ebisque and ivory modules were positively correlated with lipid peroxidation marker MDA content. Among the modules, the darkmagenta module showed strong positive correlations with antioxidant-related traits such as superoxide dismutase, guaiacol peroxidase, and glutathione s-transferase, flavonoid, and phenol content and proline accumulation, signifying its involvement in oxidative stress response and secondary metabolite biosynthesis (Fig. 10B).

KEGG pathway enrichment of darkmagenta module genes revealed significant enrichment of biosynthesis of secondary metabolites, diterpenoid biosynthesis, flavonoid biosynthesis, glutathione metabolism, and other secondary metabolite pathways, underscoring its involvement in detoxification and biosynthesis of protective compound (Fig. 10C). The gene coexpression network within this module revealed highly connected hub genes, with *GSTU6* (*Os10g0529500*), *GSTU6* (*Os10g0527400*), *GL121* (*Os12g0154700*), *GL123* (*Os12g0154900*), *APM1* (*Os09g0362800*), *AMPL1* (*Os12g0434400*), *CAD8D* (*Os09g0400400*), *C76M7* (*Os01g0561600*), and *WRKY75* (*Os11g0490900*) likely serving as candidate hub genes that coordinates metabolic response to oxidative stress and metabolite biosynthesis (Fig. 10D, Tab. S9). Overall, the darkmagenta module represented a tightly coregulated gene network that integrated antioxidant defense mechanisms and secondary metabolite biosynthesis, essential for plant adaptation to environmental stress. 

## Discussion

Deciphering the molecular and metabolic pathways that govern drought tolerance plays a crucial role to dissect effective strategies that enhance drought tolerance in rice. Despite its critical importance, studies elucidating drought tolerance mechanisms in rice during early germination remain limited. To address this gap, we carefully selected two rice accessions with contrasting drought response at the germination stage based on key morphological traits, and subsequently investigated the underlying drought adaptation mechanisms by identifying valuable genes, metabolites and pathways that contribute to enhanced drought tolerance.

Our data showed that germination rate, germination efficiency, shoot length, root length, and root number typically decrease under stress in both genotypes (Fig. 1), similar to previous results reported [34–37]. Drought adaptation forces plants to balance their resource allocation for promoting growth and enhancing stress resilience. Upland rice ecotypes, for instance, slow their growth under drought stress to conserve resources and enhance survival [36]. LY46 exhibited better resilience under PEG-induced stress with only a minor reduction in key morphological traits, whereas HGN showed a significant decrease in shoot growth and root number, though that is not the case under optimal conditions.

The morphological resilience observed in LY46 may stem from biochemical adaptations during early seed germination. Our data showed that LY46 exhibited increased antioxidant enzyme activities, along with elevated proline and phenol contents, which progressively increased over time, while soluble sugar showed a decreasing pattern (Fig. S2). Sugars and lipids serve as primary energy sources for germinating seeds until photosynthesis begins [38]. Drought stress forces plants to activate ATP-dependent tolerance mechanisms, but it disrupts starch breakdown by limiting water uptake [39]. Our KEGG enrichment analysis showed a glycolysis pathway, a crucial energy-producing pathway during seed germination, enhancement in LY46 under PEG-induced stress (Fig. 7). This finding aligns with recent studies in rice reporting significant enrichment of glycolysis-related genes under drought stress [9, 20, 40–42]. In this context, genes responsible for mobilizing stored starch, including *Os09g0457800* (encoding alpha amylase), *Os07g0452100* (encoding alpha galactosidase), and *Os07g0543300* (encoding beta amylase), were upregulated in LY46 under PEG-induced stress. Concurrently, during the early stages of stress in LY46, multiple genes associated with glycolysis and sugar metabolism were significantly upregulated. These included genes related to D-glucose (*Os02g0600400*), D-fructose (*Os06g0664200*), glyceraldehyde-3-phosphate dehydrogenase (*Os04g0459500*, *Os08g0440800*), and aldolase (*Os06g0608700*). This upregulation aligns with previous studies showing that mutations in the *OsPK1* and *OsPK5* genes [43], which disrupt monosaccharide metabolism, result in slower germination and seedling growth. The elevated expression of these genes in LY46 suggests a pivotal role of glycolysis in enhancing stress resilience.

In addition to glycolysis, KEGG enrichment revealed glutathione metabolism pathway as one of essential pathways that mediate induced stress tolerance (Fig. 7). Under optimal conditions, glutathione predominantly exists in its reduced form GSH, but drought stress increases the oxidized form GSSG, lowering the GSH to GSSG ratio and signaling oxidative stress [44]. We observed significantly higher accumulation of GSH in LY46 compared to HGN under stress (Fig. 7), consistent with glutathione’s documented role in drought [45] and salinity tolerance [46]. Additionally, LY46 exhibited higher GST activity with a steady increase over the days compared to HGN (Fig. S2). Furthermore, glutathione metabolism-related genes were regulated by a transcriptional regulatory network. TFs such as *MYB3*, *WRKY12*, and *WRKY51* have demonstrated compelling positive correlation with *GST4*, *GST5*, and *GST30*, respectively, highlighting their vital role in orchestrating the plant’s response to osmotic stress (Fig. 9, Tab. S6). These results suggested that the accumulation of GSH and other related metabolites in the glutathione metabolism pathway was possibly critical for plants to combat osmotic stress.

The phenylpropanoid biosynthesis pathway also appeared as a crucial contributor to osmotic stress tolerance in LY46 (Fig. 8). This pathway enhances stress resilience by accumulating metabolites like flavonoids and lignin. Previous studies have confirmed the crucial role of flavonoid pathway in adaptive differentiation between upland and lowland rice ecotypes [47] and in enhancing germination and promoting rice growth [48]. In our study, genes encoding F3H, PAL, 4CL, and FLS, key enzymes in the flavonoid biosynthesis pathway, exhibited increased expression under stress in LY46. Flavonoids are well known to inhibit lipid peroxidation by enhancing antioxidant defense [49]. Interestingly, naringenin, a metabolite in the flavonoid pathway that inhibits seed germination and seedling growth in other species [50] was downregulated in LY46 and may have potentially contributed to improved germination in LY46 under stress conditions (Fig. 8). Naringenin is highly accumulated in drought sensitive genotype and it requires further investigation to gain deeper insights into its transcriptional regulation in plant responses to drought stress. Like glutathione metabolism, flavonoid-related genes and metabolites were regulated by a transcriptional regulatory network, further emphasizing coordinated control of osmotic stress (Tab. S7). While glycolysis provides energy, glutathione and phenylpropanoid pathways collaborate to maintain redox balance and protect against oxidative stress, supporting successful seed germination and early growth under induced osmotic stress.

Changes in metabolite accumulation and compositions are considered as the ultimate adaptive response of plants to environmental stress. Beyond those metabolites in the above pathways, LY46 showed higher accumulation of terpenoids metabolites as compared to HGN (Tab. S5). Terpenoids were reported to possess antioxidant properties and enhance drought stress [51]. Moreover, we found that various metabolites such as peptides, glycosides, alkaloids, amines, lipids and fatty acids were significantly elevated and enriched in LY46 as compared to HGN under drought. Previous studies have indicated that these metabolites are linked to drought stress tolerance [52–56]. Additionally, LY46 accumulated higher levels of nucleotides and their derivatives, whereas low levels of this metabolite observed in HGN (Tab. S5). Increasing evidence showed nucleotides and their derivatives act as stress signaling molecule and triggering adaptive response that are involved in plant growth and development [57].

**Fig. 11:**
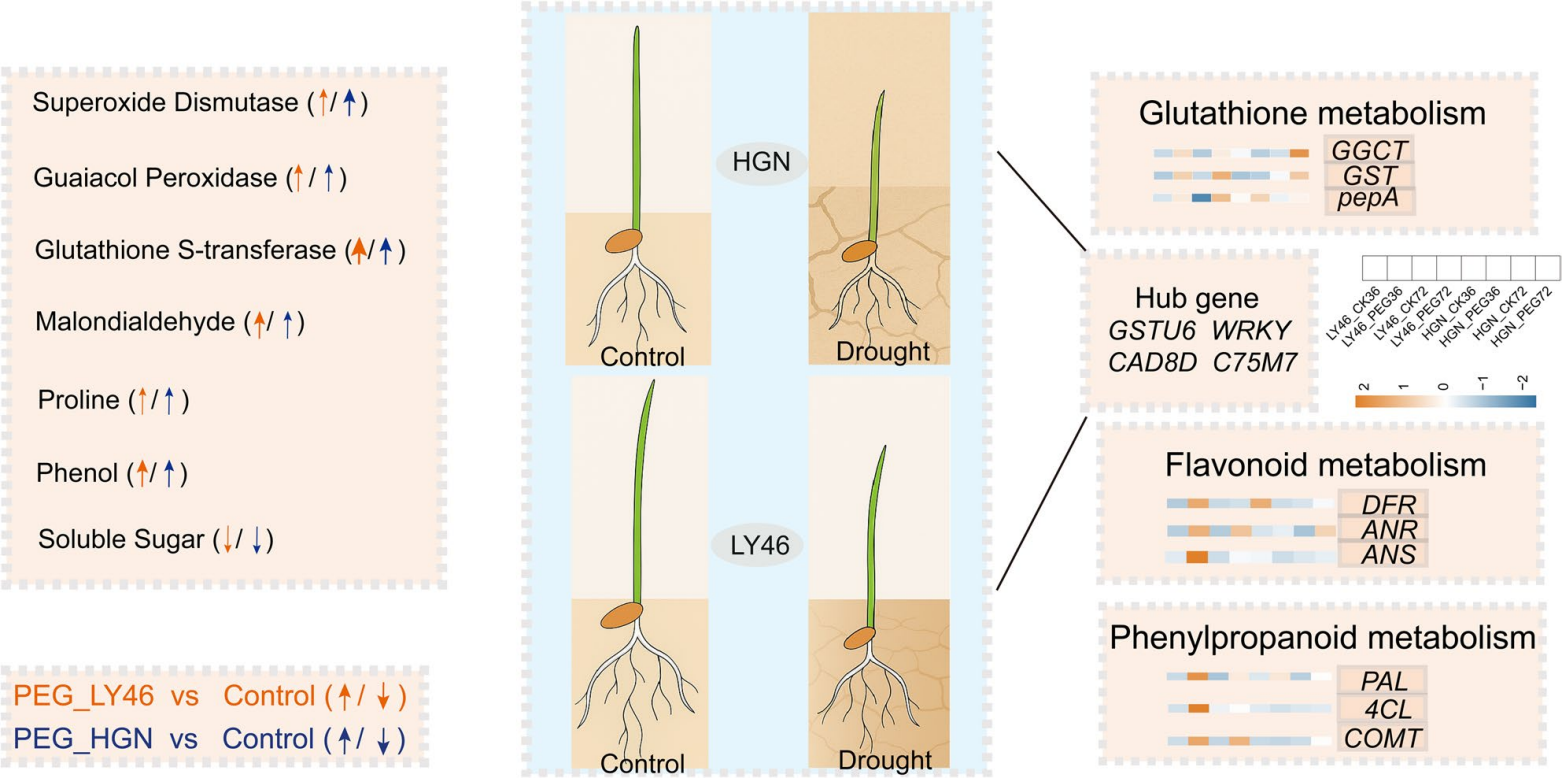
A proposed model for the enhanced induced stress tolerance of LY46

ROS are known to noticeably increase during seed germination and are further exacerbated under drought-induced osmotic stress, leading to oxidative damage if not properly managed [58]. To protect cells from oxidative damage caused by excessive ROS, plants have evolved antioxidant defense mechanisms that include both enzymatic and non-enzymatic antioxidant systems. Non-enzymatic antioxidants, such as glutathione, flavonoids, and ascorbic acid, are tightly associated with ROS scavenging under drought stress. Our biochemical assays revealed that LY46 maintained lower levels of MDA content under osmotic stress (Fig. S2). These results are consistent with previous studies that demonstrated drought-tolerant genotypes activate antioxidant defenses and accumulate osmolytes to inhibit lipid peroxidation under stress [59–64]. Complementing the biochemical test, WGCNA results revealed that specific metabolites were prominently associated within the same gene module. For instance, antioxidant enzymes such as superoxide dismutase, guaiacol peroxidase, and glutathione s-transferase, along with flavonoids, phenolic acids, and proline, were coexpressed in darkmangenta module (Fig. 8). These antioxidant enzymes and other metabolites-related genes in the module were further found to share the same hub genes network orchestrated by *GSTU6*,* WRKY75*,* GL121*,* GL123*,* C76M7*,* AMPL1*,* APM1D*,* OMTL2* and *CAD8D* (Fig. 8, Tab. S9), suggesting that these hub genes can plays a crucial role in multiple metabolic pathways in response to drought stress. *WRKY75* is an important transcription factor that plays a crucial role in transcriptional regulation of a wide range of physiological and biological processes, including drought tolerance. It has been previously demonstrated that *OsWRKY75 (Os11g0490900)* was induced by PEG, NaCl, naphthalene acetic acid (NAA), ABA and 42 °C, akin to its orthologue *AtWRKY75* in Arabidopsis [65]. This suggests that these two *WRKY* genes may play essential roles in various physiological processes in plants. In the future, more work is required to clarify how *OsWRKY75* transmits drought stress signal to regulate transcriptional regulatory network during drought stress. *GSTU6* also has been identified as a crucial hub gene and previous studies has shown that GST contributes to improved chilling and drought tolerance in rice [66]. Furthermore, the lignin biosynthesis gene *CAD8D* played a key role in regulating drought tolerance as one of the main hub gene and previous studies indicated that *CAD8D* protect water loss and improves drought tolerance, and it is likely induced by *OsERF83* [67]. The transcriptional profiling analysis demonstrated that key genes associated with the biosynthesis of these metabolites showed higher expression levels under PEG-induced stress in LY46 compared to HGN (Tab. S3). We proposed a model that illustrate observed stress tolerance in LY46. Under PEG-induced stress conditions, LY46 exhibited increased accumulations of metabolites involved in glycolysis, flavonoid and glutathione pathways, along with other metabolites, compared to HGN (Fig. 11). This elevated metabolic activity likely equipped LY46 with a robust machinery system for maintaining membrane stability and effectively scavenging ROS, thereby mitigating PEG-induced damage, ultimately contributing to enhanced drought tolerance.

Our comprehensive analysis, integrating biochemical, transcriptomic, and metabolomic data, revealed the metabolomic and molecular mechanisms underlying the enhanced osmotic stress tolerance in LY46. Hub genes and essential pathways that orchestrate osmotic stress tolerance were further explored. While these insights highlighted promising targets for boosting drought tolerance, the precise roles of these pivotal genes and metabolites require further investigation. These findings offer valuable targets for improving drought tolerance through genetic engineering or targeted application of specific metabolites.

## Supplementary Information

Supplementary Material 1: Fig S1. SEM structure and water uptake of LY46 and HGN. Fig S2. Physiological data of LY46 and HGN. Fig S3. Correlation heatmap of metabolic samples.

Supplementary Material 2: Table S1. RNA-Seq data statistics. Table S2. Unique genes annotation. Table S3. DEGs among different treatment. Table S4. Differentially expressed transcription factor among different treatment. Table S5. DEMs among different treatment. Table S6. Correlation analysis linking glutathione metabolites, genes, and key transcription factors. Table S7. Correlation analysis linking flavonoids metabolites, genes, and key transcription factors. Table S8. Darkmagneta module network nodes and edge relationships. Table S9. Top 10 hub genes names.

## Data Availability

Sequence data from this article were deposited in NCBI database (Accession No. PRJNA1313412). The data supporting the findings of this study are available in the article and its supplementary information files **.**.
