# Supplementary material for: Induction of glutathione and flavonoid biosynthesis activates antioxidant enzymes to enhance drought tolerance in rice
Source: BMC Plant Biol. 2026 Mar 9;26:683. doi: 10.1186/s12870-026-08448-3 (PMC13085399; doi:10.1186/s12870-026-08448-3)
Supplement: Supplementary file 2 — Supplementary Material 2: Table S1. RNA-Seq data statistics. Table S2. Unique genes annotation. Table S3. DEGs among different treatment. Table S4. Differentially expressed transcription factor among different treatment. Table S5. DEMs among different treatment. Table S6. Correlation analysis linking glutathione metabolites, genes, and key transcription factors. Table S7. Correlation analysis linking flavonoids metabolites, genes, and key transcription factors. Table S8. Darkmagneta module network nodes and edge relationships. Table S9. Top 10 hub genes names. [file 12870_2026_8448_MOESM2_ESM.docx]

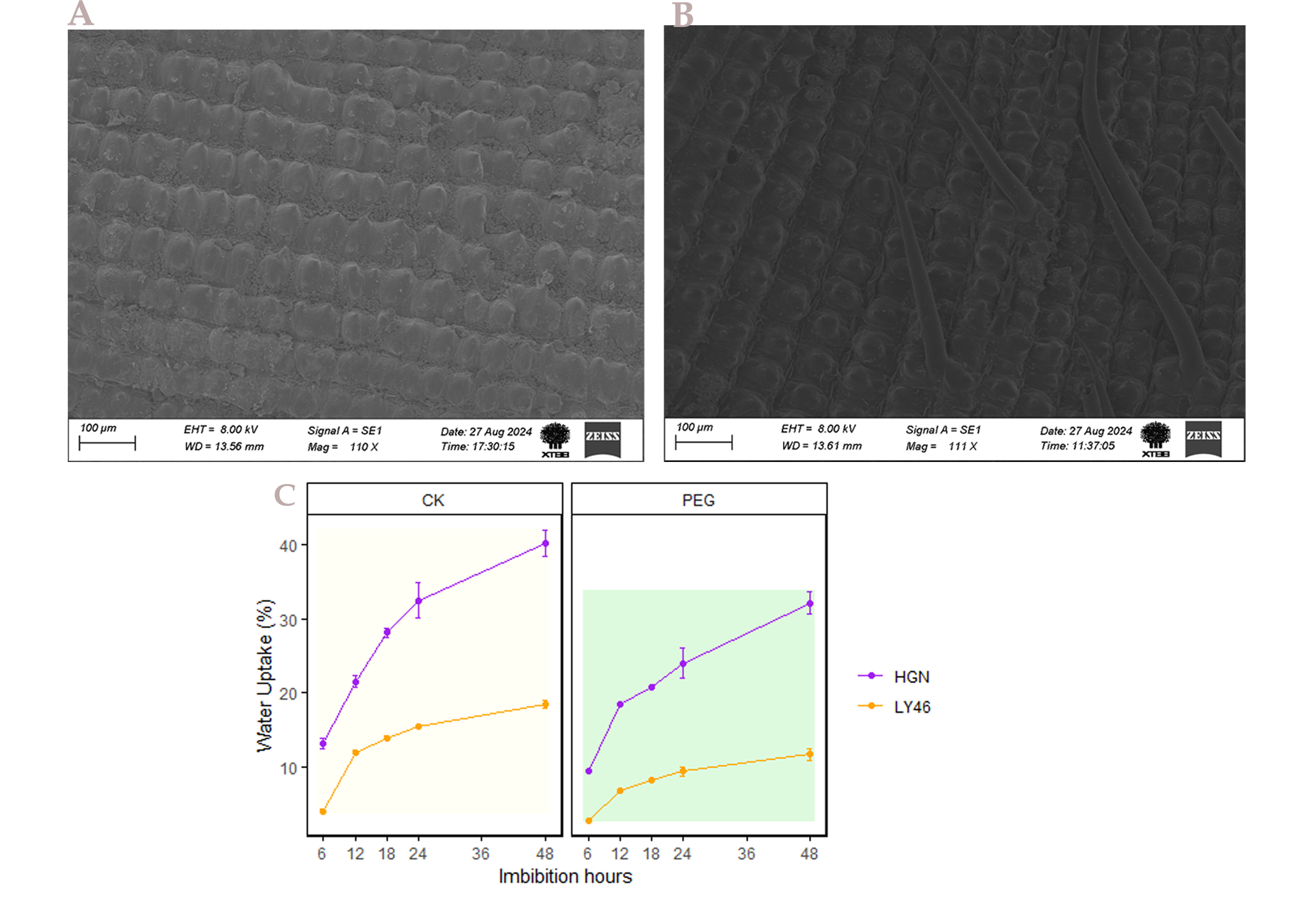


Figure S1. SEM structure and water uptake of LY46 and HGN. **(a)** HGN seed coat structure. **(b)** LY46 seed coat structure. **(c)** Water uptake, purple color indicates HGN and yellow color indicates LY46. Water uptake by seeds during imbibition was measured in triplicate, each with 10 seeds. Seeds were placed on filter paper in 9 cm petri dishes and treated with either 10 mL ddH₂O or 15% PEG-6000, then incubated at 28°C. At 6, 12, 18, 24, 36, and 48 hours after imbibition, seeds were removed, blotted dry, and weighed. Water uptake (WU%) was calculated as the percentage of water absorbed relative to the seed’s dry weight (Bhardwaj.et al., 2012).


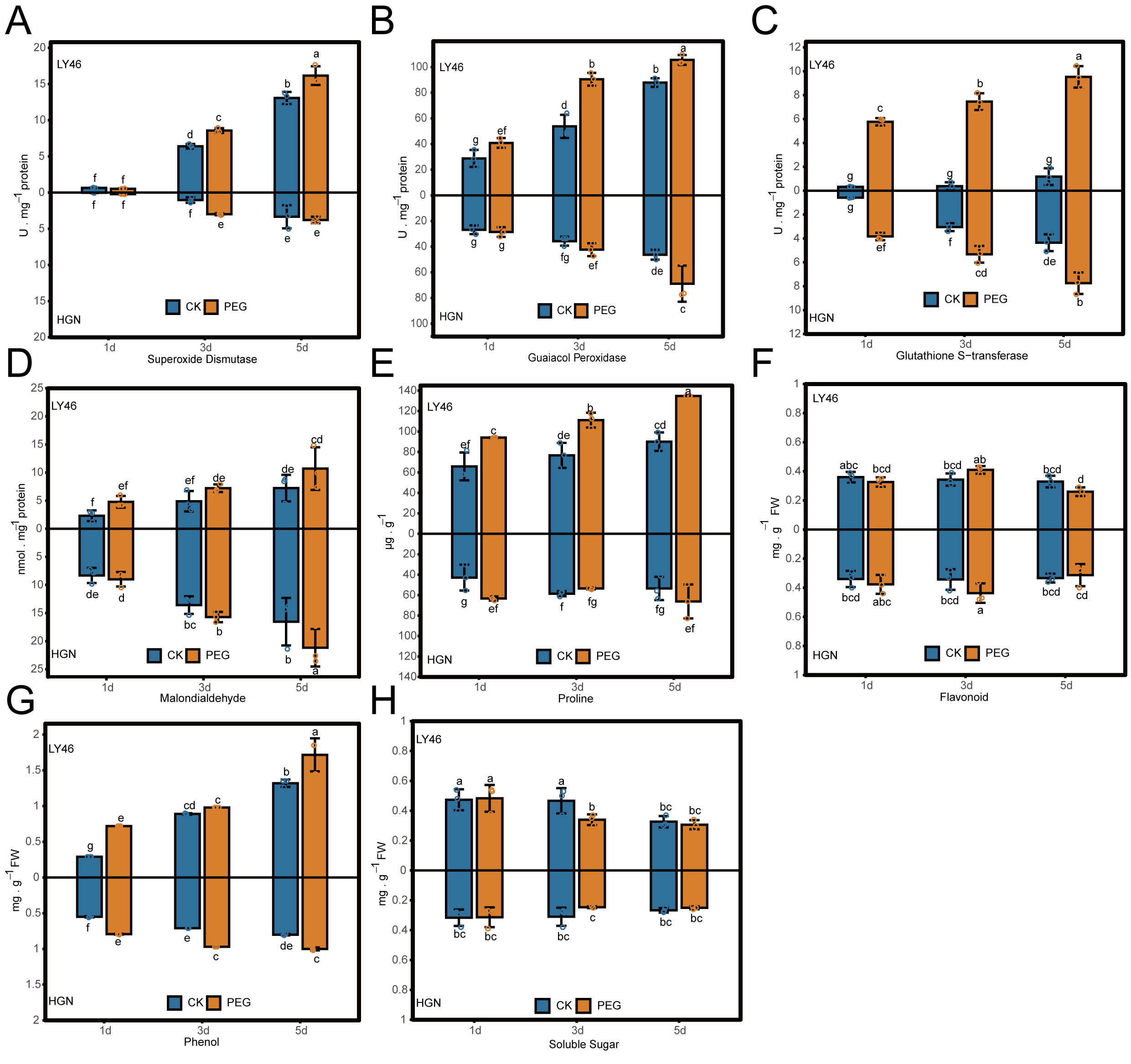


Figure S2. Physiological data of LY46 and HGN. **(a)** SOD. **(b)** GPX. **(c)** GST. **(d)** MDA. **(e)** Proline. **(f)** Flavonoid. **(g)** Phenol. **(h)** Soluble sugar. Means with the same letter are not significantly different. Blue color indicates CK and orange color indicates PEG.


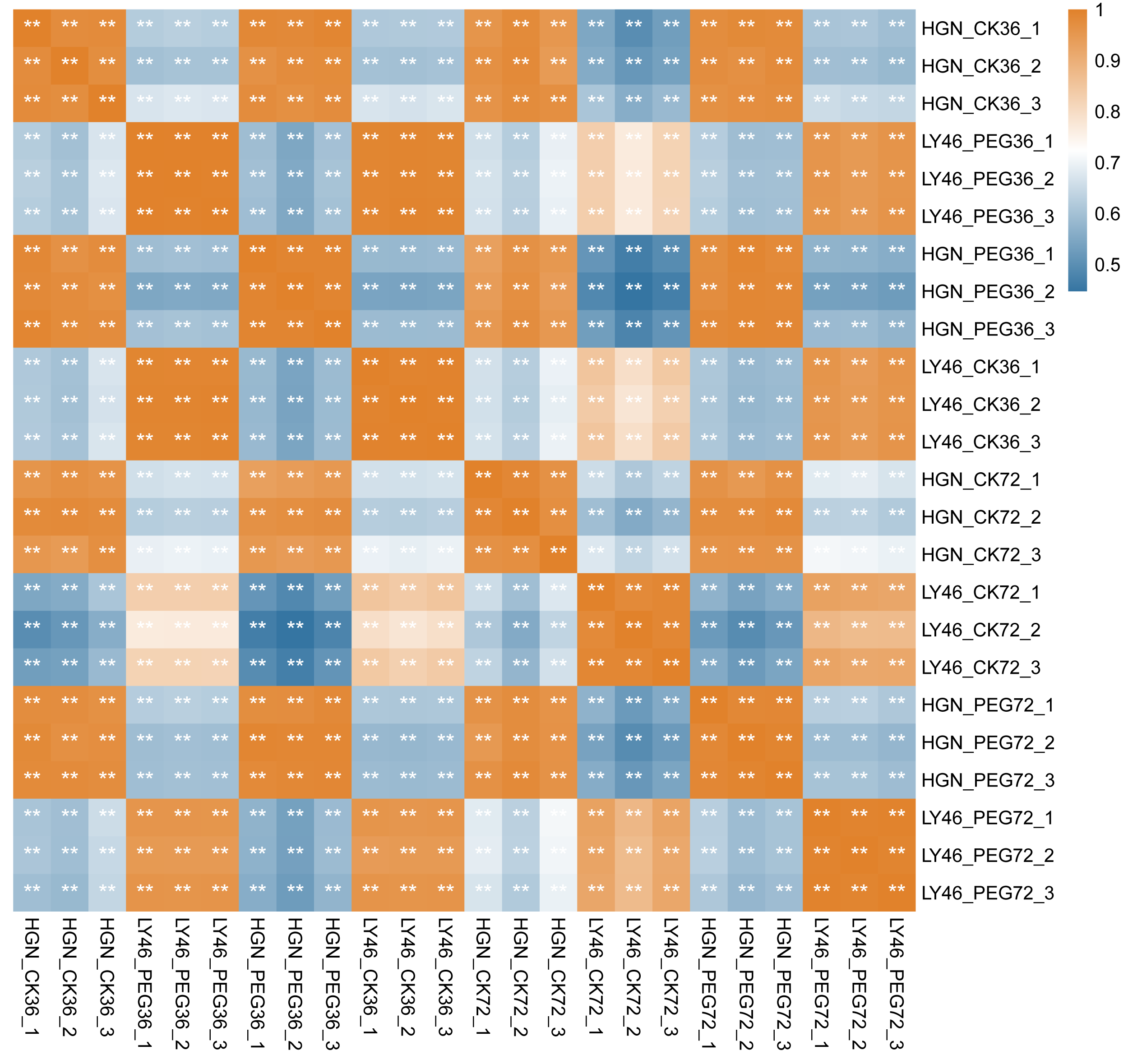


Figure S3. Correlation heatmap of metabolic samples.
